# Circ VRK1/microRNA-17/PTEN axis modulates the angiogenesis of human brain microvascular endothelial cells to affect injury induced by oxygen-glucose deprivation/reperfusion

**DOI:** 10.1186/s12868-023-00774-8

**Published:** 2023-01-27

**Authors:** Lei Yang, Hong Du, Xuejing Zhang, Bulang Gao, Dongliang Zhang, Zongrong Qiao, Xianhui Su, Tong Bao, Siqin Han

**Affiliations:** 1 Department of Neurosurgery, Shijiazhuang People’s Hospital, No.365, Jianhua South Road, Yuhua District, Shijiazhuang, 050000 Hebei People’s Republic of China; 2 Department of Cardiology, Second Hospital of Hebei Medical University, Shijiazhuang, 050000 Hebei People’s Republic of China; 3 Center of Medical Research, Shijiazhuang People’s Hospital, Shijiazhuang, 050000 Hebei People’s Republic of China; 4 Graduate School, Hebei Medical University, Shijiazhuang, 050017 Hebei People’s Republic of China

**Keywords:** Circ VRK1, microRNA-17, PTEN, PI3K/AKT pathway, Oxygen-glucose deprivation/reoxygenation

## Abstract

**Background:**

Circular RNAs (circRNAs) can act as microRNA (miRNA) sponges, thus regulating gene expression. The role of circRNAs in the process of oxygen-glucose deprivation/reoxygenation (OGD/R) is unclear. Here, we explored the mechanism underlying Circ VRK1 in human brain microvascular endothelial cells (HBMVECs) injury induced by OGD/R.

**Methods:**

The OGD/R cell model was established in HBMVECs. The microarray was applied to detect differentially expressed circRNAs, followed by subcellular fractionation assay. Colony formation assay, flow cytometry, ELISA, tube formation, Transwell and western blot assays were performed for loss-of-function assay. HE staining, TTC staining, immunohistochemistry and western blot were performed in an established mouse model. The relationships between Circ VRK1 and miR-17, and between miR-17 and PTEN were detected by bioinformatics and dual-luciferase assays. Rescue experiments were conducted in vitro and in vivo, and PI3K/AKT activity was detected by Western Blot.

**Results:**

Circ VRK1, predominantly present in the cytoplasm of cells, was upregulated in the HBMVECs exposed to OGD/R. Circ VRK1 downregulation decreased proliferation, migration, tube formation, inflammatory factors and oxidative stress, while increased apoptosis in HBMVECs. Moreover, Circ VRK1 silencing reduced neurological damage, cerebral infarct size, CD34-positive cell counts and VEGF expression in mice. Circ VRK1 mediated PTEN expression and the PI3K/AKT pathway by targeting miR-17. Deletion of miR-17 inhibited the effects of Circ VRK1 siRNA, and silencing of PTEN suppressed the effects of miR-17 inhibitor.

**Conclusion:**

Circ VRK1 was upregulated during OGD/R. Circ VRK1 downregulation regulates PTEN expression by targeting miR-17, thereby promoting PI3K/AKT pathway activity to alleviate OGD/R injury.

**Supplementary Information:**

The online version contains supplementary material available at 10.1186/s12868-023-00774-8.

## Introduction

Ischemia refers to the interruption of metabolic fuel and oxygen supply to maintain cellular oxidative metabolism, and restoration of oxygen upon reperfusion of ischemic tissue activates oxidative stress which induces the reperfusion injury cascade, leading to injury and death of cells and tissues [1]. However, efficient clinical drugs for the treatment of cerebral ischemia-reperfusion injury are still absent. Angiogenesis, the growth of new blood vessels, could be termed as a natural defense mechanism, which is beneficial to reintroduce oxygen and nutrient delivery to the affected brain tissues [2]. Therefore, an in-depth understanding of the detailed mechanisms of angiogenesis is of great importance for developing effective treatments for cerebral ischemia-reperfusion injury.

The emerging appreciation of the roles of circular RNAs (circRNAs) has given rise to a novel perception on our understanding of cellular physiology and disease pathogenesis [3]. CircRNAs are characterized by a covalent bond linking the 5′ and 3′ ends, and they have plentiful potential functions, such as serving as microRNA (miRNA) sponges or binding to RNA-associated proteins [4]. There are 65,731 and 15,849 circRNAs candidates detected in human and mouse brain samples, respectively [5]. CircRNAs are vital regulators in the pathogenesis of cerebral ischemia-reperfusion injury and consequently might be possible therapeutic targets for cerebral ischemia diseases [6]. Human brain microvascular endothelial cells (HBMVECs) contribute drastically to integrity and function of the brain vasculature, and oxygen and nutrient deprivation may trigger HBMVEC dysfunction [7]. However, how oxygen-glucose shortage affects circRNAs in HBMVECs is unknown. Here, we used circRNA microarrays to measure the changes of circRNAs in HBMVECs subjected to oxygen-glucose deprivation and reoxygenation (OGD/R) treatment or not. CircRNA VRK serine/threonine kinase 1 (circ VRK1) was identified to be the most significantly upregulated in HBMVECs treated with OGD/R. Located at chromosome 14 from 97,312,431 to 97,327,072, Circ VRK1 was found reduced in breast cancer tissues and linked to reduced tumor stage and better survival outcome [8]. However, its role in the brain remains largely unclear. Interestingly, Circ-ITCH has been reported to protect myocardial cells from injuries evoked by H_2_O_2_ by interacting with miR-17-5p [9]. More importantly, miR-17-5p elevated the endothelialization of endothelial progenitor cells to expedite the vascular repair of aneurysm by regulating PTEN-mediated PI3K/AKT signaling [10]. In addition, hsa-miR-17 has been linked to the development of intracranial aneurysm in relation to the PI3K/AKT signaling pathway [11]. Therefore, in this study, we hypothesized that Circ VRK1 targets miR-17 to modulate PTEN-mediated PI3K/AKT signaling, thus involving in the development of cerebral ischemia-reperfusion injury.

## Materials and methods

### Cell culture and infection

HBMVECs were purchased from Angio-Proteomie (Boston, MA, USA). Cells were cultured in endothelial cell culture medium (ScienCell, Carlsbad, CA, USA) plus endothelial cell growth supplement and 5% fetal bovine serum (FBS) with 5% CO_2_ and 95% air. HBMVECs were exposed to 1% O_2_ + 5% CO_2_ + 94% N_2_ in glucose-free DMEM (Gibco, Carlsbad, CA, USA) under anoxic conditions for 2 h, 4 h, and 8 h, respectively. Afterwards, the cells were reoxygenated in complete medium for 24 h to obtain OGD/R cells. The control cells were cultured normally in 5% CO_2_ and 95% air.

Circ VRK1 siRNA, miR-17 inhibitor and PTEN silencing lentiviral vector were provided by Shanghai Sangon Biological Engineering Technology & Services Co., Ltd. (Shanghai, China). HBMVECs were seeded into 10-cm dishes (5 × 10^6^ cells/dish) and incubated for 12 h prior to lentivirus infection. The lentiviruses were then added to a culture dish with a multiplicity of infection of 10 to infect the cells for 24 h. The infected HBMVECs were used for the experiments.

### Colorimetric assays

The CyQUANT™ lactate dehydrogenase (LDH) cytotoxicity test kit (Thermo Fisher Scientific Inc., Waltham, MA, USA) was used to assess the apoptotic cell or cell injury. Cells were seeded into 96-well cell culture plates, and the wells were divided into the following groups: cell-free culture medium wells (background blank), untreated control wells (sample control wells), untreated cell wells for subsequent lysis (sample maximum enzyme activity control wells), and OGD/R-treated cell wells (sample wells). The cell culture plate was removed from the cell incubator 1 h before the assay, and 10% LDH release reagent was added to the sample maximum enzyme activity control wells. After incubation in the cell culture incubator for 2 h, the cells were centrifuged at 400 g for 5 min. A total of 120 µL cell supernatant was added to 60 µL LDH assay working solution and incubated for 30 min at room temperature (about 25℃) in the dark. Then, the optical density (OD) was read at 490 nm, cytotoxicity = (OD value of treated sample−OD value of control sample)/(OD value of maximum cellular enzyme activity−OD value of control sample).

### circRNA microarray analysis

RNA from each sample was subjected to microarray analysis and hybridization as per the manufacturer’s protocol (Arraystar Inc., Rockville, MD, USA). Sample preparation and microarray hybridization were performed according to the standard protocol. Total cellular RNA was detached with Rnase R (Epicentre Technologies, Madison, WI, USA) to remove linear RNA and abundant circRNA. The enriched circRNA was then amplified, and the labeled cRNA was hybridized to an Arraystar circRNA array (8 × 15k, Arraystar) using a random primer method (Arraystar Super RNA Labeling Kit; Arraystar). The arrays were scanned with an Agilent G2505C scanner (Agilent Technologies, Santa Clara, CA, USA), and the acquired array images were analyzed using feature extraction software (11.0.1.1, Agilent). The R package was used for quantile normalization and subsequent data processing, and circRNAs that were differentially expressed between the two groups (|Foldchange| > 2 and *p* < 0.01) were screened using a heatmap.

### Real-time PCR

Total RNA was isolated from HBMVECs and brain infarct tissues using TRIzol reagent (Themo Fisher). Total RNA was reversely transcribed using a one-step method RT-qPCR kit (Sangon Biotech, Shanghai, China) and then amplified with PCR primers on a CFX96 Touch Real-Time PCR Detection System (Bio-Rad, Inc., Hercules, CA, USA). Expression of the target genes were normalized to glyceraldehyde-3-phosphate dehydrogenase (GAPDH) or U6 using the 2^−ΔΔCt^ method. The primer sequences are shown in Table 1.

**Table 1:** PCR primers sequences for gene

Gene name	Primers
hsa-Circ VRK1	Forward:5′-TACCAACGAGCTGCAAAACC-3′
	Reverse: 5′-TCACTCCCAAAGCGATCCAT-3′
mmu-Circ VRK1	Forward:5′-GGCGGACACAAATTCTTCCA-3′
	Reverse: 5′-ACTCCCAAAGCGGTCCATTA-3′
hsa-miR-17	Forward:5′-TGCTTACAGTGCAGGTAG-3′
	Reverse: 5′-GAACATGTCTGCGTATCTC-3′
mmu-miR-17	Forward:5′-AAGTGCTTACAGTGCAGGT-3′
	Reverse: 5′-GAACATGTCTGCGTATCTC-3′
(hsa and mmu) PTEN	Forward:5′-CATGTTGCAGCAATTCACTG-3′
	Reverse: 5′-CTTGTGAAACAACAGTGCCA-3′
(hsa and mmu)VEGF	Forward: 5′-TTGCCTTGCTGCTCTACCTCCA − 3′
	Reverse: 5′-GATGGCAGTAGCTGCGCTGATA − 3′
(hsa and mmu) U6	Forward:5′-CTCGCTTCGGCAGCACATA-3′
	Reverse: 5′-AACGCTTCACGAATTTGCG-3′
(hsa and mmu) GAPDH	Forward: 5′-CATCACTGCCACCCAGAAGACTG-3′
	Reverse: 5′-ATGCCAGTGAGCTTCCCGTTCAG-3′

*hsa* homo sapiens; *mmu* mus musculus; *circRNAs* circular RNAs; *miRNA* microRNA; *PTEN* phosphatase and tensin homolog; *VEGF* vascular endothelial growth factor; *GAPDH* glyceraldehyde-3-phosphate dehydrogenase

### Subcellular fractionation

The PARIS Kit (Thermo Fisher) was used to isolate RNA from the cytoplasm and nucleus. Cells were resuspended using cell fractionation buffer, centrifuged at 1,500 rpm for 10 min at 4 °C, and the supernatant and precipitate were separated. Cell disruption buffer was added to the precipitate, and the supernatant and the precipitate suspension were mixed with 2× lysis/binding solution and added to the adsorption column, followed by three washes with wash solution to purify the RNA. U6, GAPDH and Circ VRK1 expression in the nucleus and cytoplasm of cells was subsequently measured.

### Cell counting kit-8 (CCK8) assay

The effect of OGD/R on cell viability was measured using CCK8. Cells were detached with 0.25% trypsin and centrifuged at 1,000 rpm for 3 min. The cells were resuspended in phosphate-buffered saline and plated in 96-well plates at 5 × 10^3^ cells/well. After the exposure to 1% O_2_ + 5% CO_2_ + 94% N_2_ under hypoxic conditions for 2 h, 4 h, and 8 h, respectively, the cells were reoxygenated in complete medium for 24 h to obtain OGD/R cells and incubated again with 10 µL CCK8 solution (Abmole Bioscience Inc., Houston, TX, USA) for 2 h. The cell viability was then assessed by recording the OD value of each well using a microplate reader (450 nm, Bio-Rad).

### Colony formation assay

The detached cells were seeded in 6-well plates at 5 × 10^3^ cells/well, and the medium was refreshed at an interval of three days. The culture was terminated after 10 days of incubation until colonies could be observed. The colonies were fixed in 5 mL methanol for 15 min and stained with 0.5% crystal violet for 20 min. The number of colonies with more than 10 cells was counted under the microscope [12].

### Measurement of cell apoptosis

Flow cytometry combined with an Annexin V-allophycocyanin (APC) and PI fluorescent double-stained apoptosis detection kit (Procell, Wuhan, Hubei, China) was used to detect apoptosis in HBMVECs. The cells were harvested after OGD/R and lentiviral vector treatment, and then 5 × 10^4^ cells were double stained with Annexin V-APC and PI for 5 min. Annexin V and PI double positive cells were sorted in a flow cytometer, and the percentage of positive cells was analyzed using the FlowJO software.

### Measurement of inflammatory and oxidative stress factors

Cells were lysed with 100 µL pre-cooled RIPA lysis buffer for 15 min at 4 °C, and the supernatant was collected after centrifugation at 2500 r/min for 15 min at 4 °C. Enzyme-linked immunosorbent assay (ELISA) kits were used for the analysis of TNF-α (SEKH-0047), IL-1β (SEKH-0002), and IL-6 (SEKH-0013). Finally, the OD value at 450 nm was measured with a microplate reader (Bio-Rad), which was converted to concentration (pg/mL) using a standard calibration curve. The oxidative stress factors SOD (BC0170) and MDA (BC0020) were detected by visible spectrophotometric method. ROS (CA1410) was detected using the fluorescent probe dichloro-dihydro-fluorescein diacetate (DCFH-DA). Assay kits were provided by Beijing Solabio Life Sciences Co., Ltd. (Beijing, China).

### Measurement of cell migration

Transwell assays were used to measure cell migration by plating 1 × 10^4^ HBMVECs into the apical chamber of a Transwell chamber (Corning Glass Works, Corning, N.Y., USA). Serum-free DMEM was supplemented to the apical chamber of the Transwell, and DMEM containing 10% FBS was supplemented to the basolateral chamber of the Transwell. The filters (Millipore Corp, Billerica, MA, USA) were removed after 48 h of incubation at 37 °C, and the cells were removed from the surface of the filters using cotton swabs. The cells were stained with 1% crystal violet and assessed under a Nikon Eclipse Ti inverted microscope (Nikon Instruments Inc., Melville, NY, USA).

### Tube formation assay

To assess the effect of genes on angiogenesis, we performed a tube formation assay by adding 25 µL pre-cooled Matrigel (Corning) diluted in DMEM at 1:1 to 96-well plates and placed at 37 °C for 30 min. Cells were added to 96-well plates at 2 × 10^4^ cells/well and incubated for 6 h, followed by the observation of tube formation in the Matrigel under a Nikon Eclipse Ti inverted microscope (Nikon). The angiogenesis was evaluated on the basis of vessels area and total vessel length using the AngioTool, a computational tool for quantitative analysis of vascular networks [13].

### Western blot

HBMVECs and brain tissues were harvested and lysed in RIPA lysis buffer (Sigma-Aldrich). The lysate was centrifuged at 11,000 ×*g* for 2 min at 4 °C to collect the proteins. The proteins were separated via 10% sodium dodecyl sulfate-polyacrylamide gel electrophoresis and electrophoretically transferred to polyvinylidene fluoride membranes (Millipore, pre-cut size was 6.6 × 8.5 cm). The membranes were blocked with 5% skim milk for 2 h and hybridized with primary antibodies to VEGF (1:1000, sc-7269, Santa Cruz Biotechnology Inc., Santa Cruz, CA, USA), PI3K (1:2000, ab86714, Abcam, Cambridge, UK), p-PI3K (phospho Y607, 1:1000, ab182651, Abcam), AKT (1:800, 10176-2-AP, ProteinTech Group, Chicago, IL, USA), p-AKT (1:500, 66444-1-Ig, ProteinTech Group) and GAPDH (1:1000, ab8245, Abcam) overnight at 4 °C. The membrane was washed and incubated with secondary antibodies goat anti-mouse IgG H&L (HRP) (1:5000, ab205719, Abcam) or goat anti-rabbit IgG H&L (HRP) (1:5000, ab6721, Abcam). The blots were then visualized by Novex™ enhanced chemiluminescence reagents (Thermo Scientific), and the results were normalized to GAPDH.

### Establishment of an animal model

This study was approved by the Animal Care and Ethics Committee of Shijiazhuang People’s Hospital and carried out in compliance with the Animals in Research: Reporting In vivo Experiments guidelines. All animal procedures were in accordance with the Guide for the Care and Use of Laboratory Animals issued by the National Institutes of Health (NIH, Bethesda, Maryland, USA). Great efforts were made to minimize the pain of animals. The male C57BL/6 mice (n = 96, body weight 22.5 ± 24 g, Shanghai Laboratory Animal Research Center, Shanghai, China) were anesthetized by intraperitoneal injection of 50 mg/kg sodium pentobarbital. The external carotid artery (ECA) was identified by incising the ventral midline under the surgical microscope, and the distal ECA was ligated. A nylon suture with a length of 3.0 cm and a diameter of 0.104 mm was introduced into the lumen of the ECA to tighten the silk suture around the ECA to secure the intraluminal nylon suture. The nylon suture was advanced from the ECA to the middle cerebral artery (MCA), and the decline in local cerebral blood flow was monitored using a laser Doppler flowmeter to confirm MCA occlusion. The incisions were closed with 3 − 0 silk sutures, and the mice were then carefully placed in recovery cages. The mice were re-anesthetized just before the end of the occlusion period, and the incision was reopened by removing the closed sutures. The obstructing sutures were removed from the ECA to restore perfusion. One week after recovery, the mice were grasped by the tail and observed to test for behavioral indications of infarction by turning to either side. Neurological symptoms in animals were assessed using the Zea Longa 6-point system [14]. For the sham group, no filament blocking was performed, and the rest of the steps were the same as the model development steps. After successful establishment of the animal model, 5 µL lentiviral vector was injected into the lateral ventricles to knock down the gene expression, and subsequent experiments were performed one week after lentiviral vector injection.

### Tripheny tetrazolium chloride (TTC) staining

The mice were euthanized by injecting 120 mg/kg sodium pentobarbital, and then the hearts were perfused with saline. The brain tissues were harvested and cut into sections with a thickness of about 2 mm along the coronal plane. The brain tissue sections were placed in 2% TTC staining solution (Solarbio), incubated at 37 ℃ for 30 min, and then photographed. Calculation of the infarct volume was conducted using Image Pro where normal brain tissue is red and the infarcted area is pale.

### Hematoxylin-eosin (HE) staining

The brain tissues were fixed with 4% paraformaldehyde overnight, followed by conventional sectioning, embedding and staining. The tissues were dewaxed in xylene, soaked in gradient concentration ethanol for 5 min, stained with hematoxylin staining solution (Solarbio) for 5 min, and soaked in 1% hydrochloric acid ethanol for 3 s. Then, the tissue was stained with 5% eosin (Solarbio) for 3 min, dehydrated, and sealed with neutral gum. Five randomly selected fields of view were photographed under an inverted microscope (Eclipse Ti, Nikon) to observe the tissue sections.

### Immunohistochemistry (IHC)

After obtaining mouse brain tissues for conventional paraffin-embedding, the tissues were cut into 4-µm-thick histological sections, dewaxed with xylene, hydrated with gradient concentration ethanol, and boiled for 10 min at 100 °C in citric acid buffer (pH = 6.0) for antigen extraction. Sections were incubated with antibody to CD34 (1:500, MA1-10202, Thermo Fisher) at 4 °C overnight, followed by incubation with horseradish peroxidase-coupled secondary antibody (1:1,000, ab205719, Abcam) for 120 min at room temperature. The color development reaction was performed using DAB, and the nuclei were counter-stained by hematoxylin. Positive staining intensity and area percentage were scored semi-quantitatively using IHC Profiler [15] in the Image J software. The final IHC scores = percentage contibution of high positive × 3 + percentage contibution of positive × 2 + percentage contibution of low positive × 1.

### Luciferase reporter gene assay

The localization and sequence of circ_0000566 (circ VRK1) were analyzed using circBase (http://circbase.org/), and the downstream miRNAs of circ VRK1 and the binding sites between circ VRK1 and miR-17, miR-17 and PTEN were obtained in StarBase. The 3′untranslated region (3′UTR) containing the wild-type (WT) of the PTEN binding sites with miR-17 or mutant (MT) was subcloned into the restriction site of the pmiR-RB-REPORT vector (RiboBio Co., Ltd., Guangzhou, Guangdong, China). The circVRK1 sequence containing the wild-type or mutant miR-17 binding site was inserted into the restriction site of the pmiR-RB-REPORT vector. The luciferase reporter vector was co-transfected into 293T (American Type Culture Collection, Manassas, VA, USA) cells using Lipofectamine 3000 (Life Technologies, Carlsbad, CA, USA) together with miR-17 control or miR-17 inhibitor. Forty-eight hours after transfection, luciferase activity was detected by the Dual-Luciferase Reporter Assay System (Promega, Madison, WI, USA) as per the manufacturer’s instructions.

### Statistics

Statistical analysis was conducted using SPSS 22.0 software (IBM Corp. Armonk, N.Y., USA). Data were exhibited as then mean ± standard deviation (SD), and all experiments were repeated three times. Comparison among three or above groups was determined by one-way or two-way ANOVA followed by Tukey’s multiple comparison test; comparison between two groups was determined by unpaired *t* test. Statistical significance can be accepted when *p* < 0.05.

## Results

### OGD/R causes high expression of Circ VRK1 in HBMVECs

**Fig. 1:**
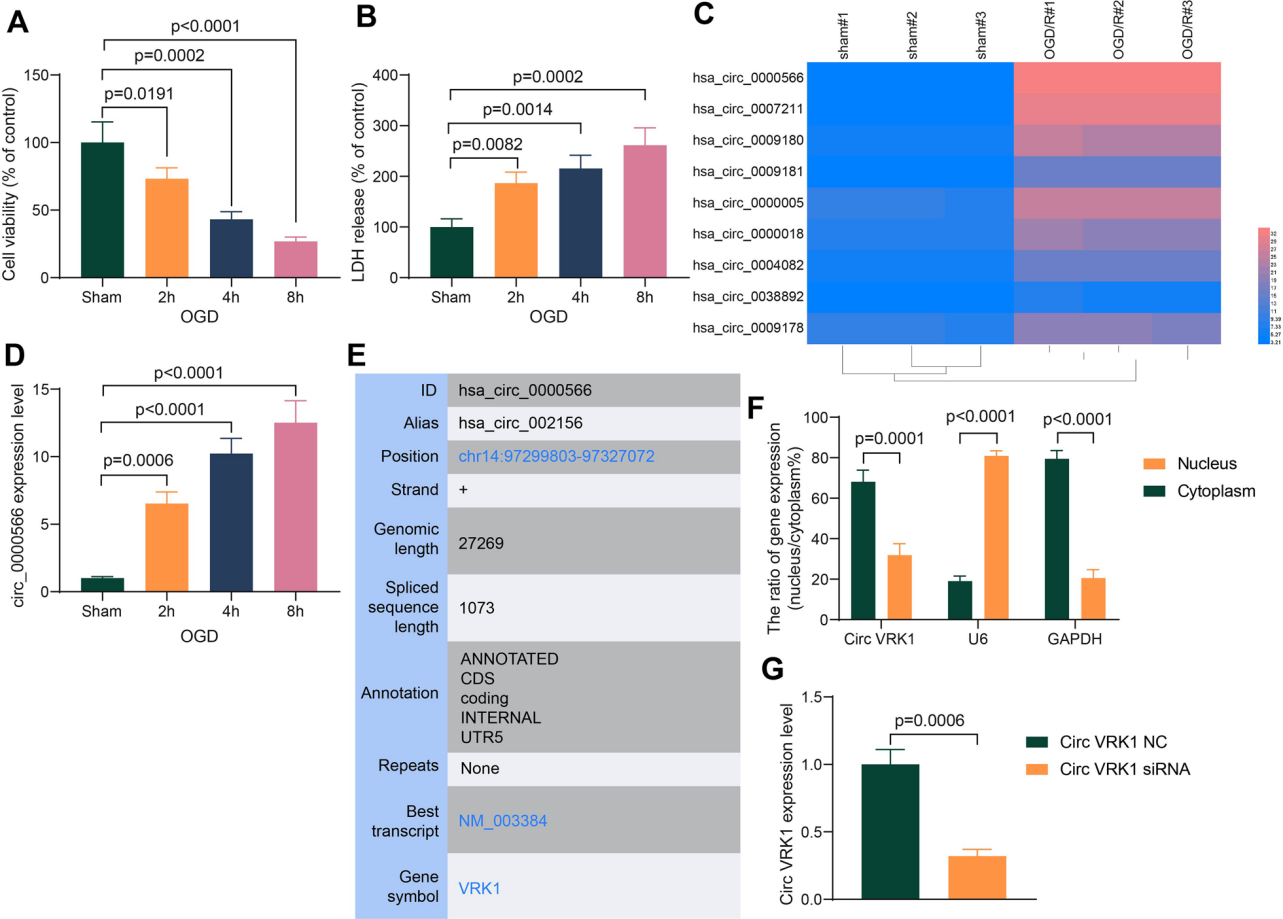
OGD/R treatment decreases HBMVEC viability and upregulates Circ VRK1 expression. **A** the viability of HBMVECs exposed to OGD for 2 h, 4 h and 8 h detected by CCK8 assay. **B** The cytotoxicity of HBMVECs exposed to OGD for 2 h, 4 h and 8 h measured by LDH release assay. **C** circRNA microarray analysis of differentially expressed circRNAs in cells exposed to OGD/R. **D** circ_0000566 expression changes in HBMVECs exposed to OGD for 2 h, 4 h and 8 h by RT-qPCR. **E** The genome position and source genes of circ_0000566 queried in circBase. **F** the localization of Circ VRK1 in HBMVECs determined by subcellular fractionation. **G** PCR validation of Circ VRK1 siRNA transfection efficiency in HBMVEC. Error bars represented the mean ± SD from 3 independent experiments. Unpaired *t* test (**G**) and one-way (**A**–**D**) or two-way ANOVA (**F**) were used

To investigate the effect of OGD/R on cell viability, we first established an OGD/R model. The cell viability was decreased in a time-dependent manner during OGD/R (Fig. 1A). Meanwhile, the detection of cytotoxic changes revealed that the content of LDH released from cells was increased sequentially with the time advancement of OGD/R (Fig. 1B). These results confirmed the damaging effect of OGD/R on cells in vitro. Next, we performed circRNA microarrays to detect changes in circRNA expression in HBMVECs after 8 h of OGD/R. Nine circRNAs were differentially highly expressed after cells were treated with OGD/R (Fig. 1 C). We observed that the expression foldchange of circ_0000566 was significantly larger after OGD/R treatment than other eight circRNAs. qPCR was then used to verify the results of microarray analysis, and circ_0000566 was found to be significantly higher after OGD/R treatment (Fig. 1D).

The mechanism of circ_0000566 in HBMVECs after OGD exposure was investigated. CircBase (http://circbase.org/) revealed that circ_0000566 is derived from the VRK serine/threonine kinase 1 (VRK1) with a spliced sequence length of 1073 bp (Fig. 1E). Circ VRK1 was principally sublocalized in the cytoplasm of HBMVECs by subcellular fractionation assays (Fig. 1F). Circ VRK1 siRNA was then transfected into cells to downregulate the expression of Circ VRK1 in cells, and the transfection efficiency of siRNA was confirmed by PCR (Fig. 1G).

### Circ VRK1 affects OGD/R-induced changes in HBMVECs

**Fig. 2:**
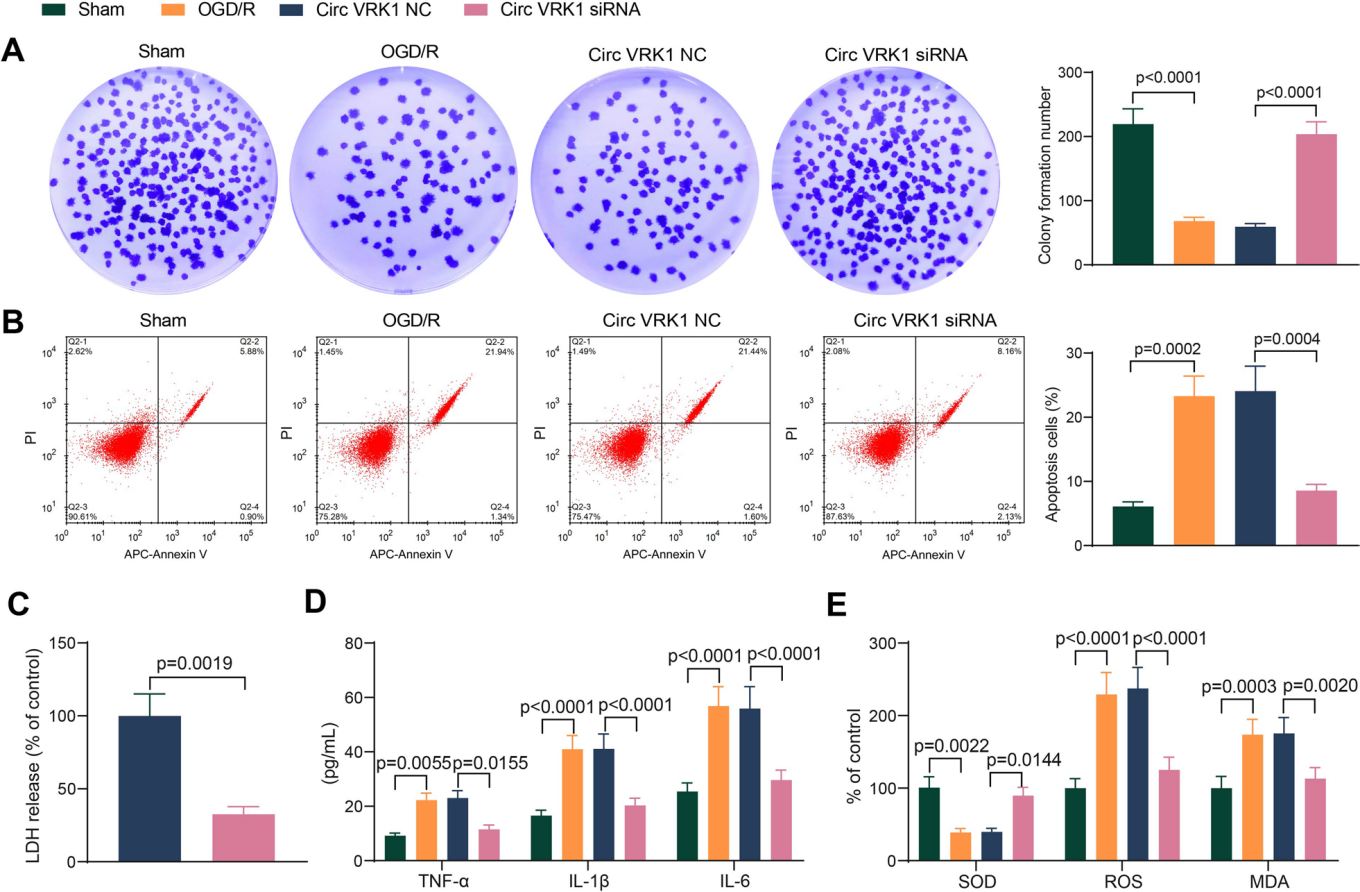
Silencing of Circ VRK1 reduces apoptosis and promotes viability in OGD/R-treated HBMVECs. HBMVECs were infected with lentiviral vectors harboring Circ VRK1 NC or Circ VRK1 siRNA and exposed to OGD/R. **A** proliferation of HBMVECs examined by colony formation assay. **B** apoptosis of HBMVECs examined by flow cytometry. **C** changes in LDH levels released from HBMVECs examined by cytotoxicity assay. **D** levels of inflammatory factors in HBMVECs examined by ELISA kits. **E** oxidative stress factor levels in HBMVECs measured by spectrophotometry and fluorescent probes. Error bars represented the mean ± SD from 3 independent experiments. Unpaired *t* test (**C**) and one-way (**A**, **B**) or two-way ANOVA (**D**, **E**) were used

The results of colony formation assays exhibited that OGD/R significantly led to a decrease in the number of colony formation of HBMVECs, while Circ VRK1 siRNA significantly induced the proliferation of the cells (Fig. 2A). Flow cytometry showed that OGD/R significantly enhanced apoptosis in HBMVECs, while Circ VRK1 siRNA resulted in a relative decrease in apoptosis (Fig. 2B). Cytotoxicity was also shown to be attenuated by downregulation of Circ VRK1 (Fig. 2C). The levels of TNF-α, IL-1β, IL-6, SOD, ROS, and MDA were measured in cells. It was revealed that OGD/R treatment increased the inflammatory response in cells, while downregulation of Circ VRK1 suppressed inflammatory factors in cells (Fig. 2D). Meanwhile, we found that the oxidative stress markers ROS and MDA were found to be significantly increased and SOD was significantly decreased under OGD/R induction, whereas ROS and MDA was significantly reduced and SOD content was augmented under the effect of Circ VRK1 siRNA (Fig. 2E). This experiment showed that OGD/R repressed the cellular activity of HBMVECs, while the downregulation of Circ VRK1 was able to slow down this change, suggesting that OGD/R regulates the cellular activity of HBMVECs through Circ VRK1.

### Circ VRK1 knockdown promotes angiogenesis of HBMVECs exposed to OGD/R

**Fig. 3:**
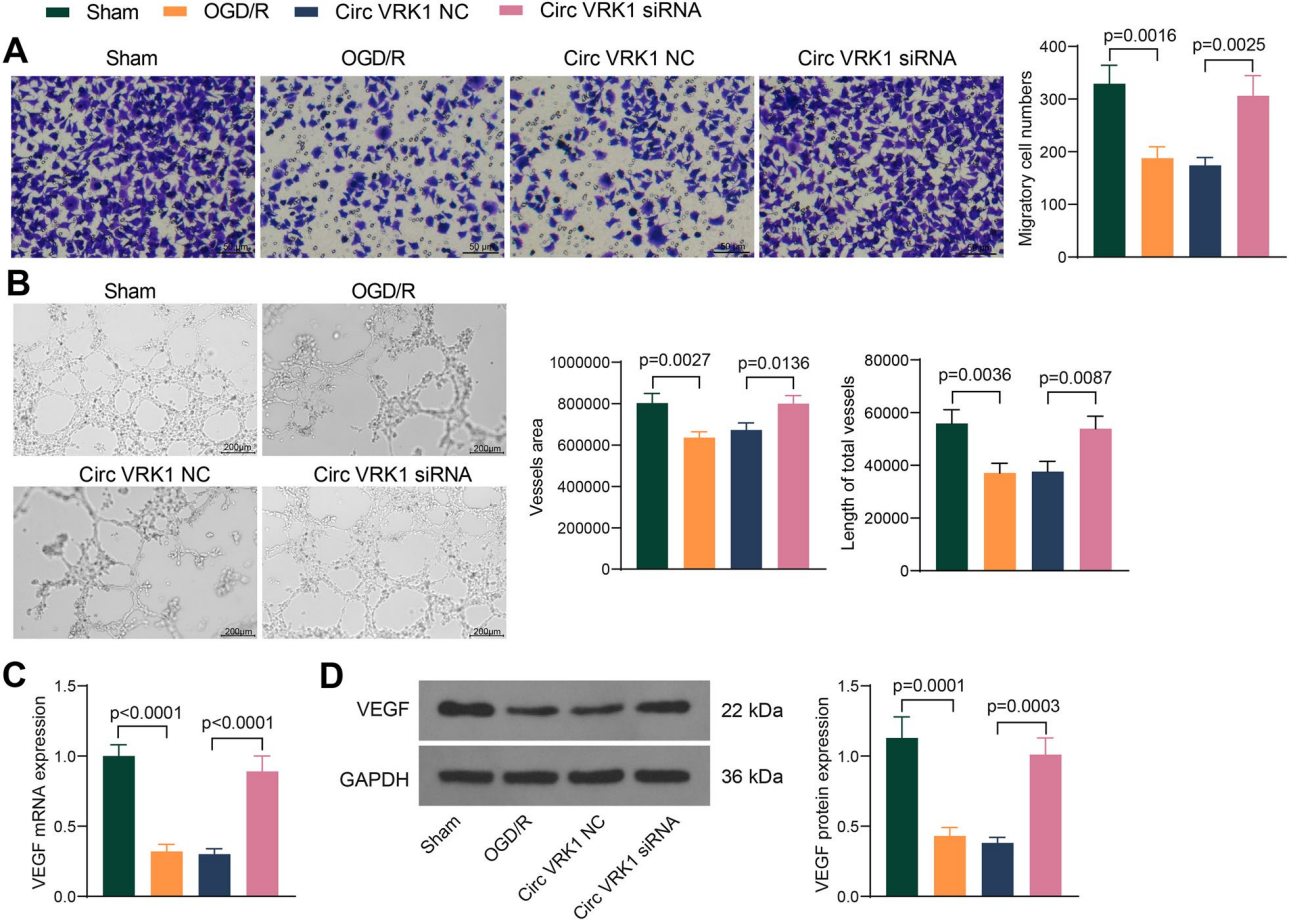
Silencing of Circ VRK1 induces angiogenesis and migration of HBMVECs. HBMVECs were infected with lentiviral vectors harboring Circ VRK1 NC or Circ VRK1 siRNA and exposed to OGD/R. **A** the migration of HBMVECs assessed by Transwell assay. **B** vessels area and total vessel length in HBMVECs assessed by tube formation assay. **C** VEGF mRNA expression in HBMVECs by RT-qPCR. **D** Representative pictures of western blotting for VEGF protein expression in HBMVECs, and all replicates are presented in the Additional file 1 (images of adequate length are not provided). Error bars represented the mean ± SD from 3 independent experiments. One-way ANOVA were used

Tube formation and migration assays were performed to investigate the effect of Circ VRK1 on angiogenesis in vitro. OGD/R resulted in a notable decrease in migration capacity of HBMVECs, whereas downregulation of Circ VRK1 significantly promoted cell migration (Fig. 3A). Consistently, vessels area and total vessels length were suppressed in OGD/R-treated cells, while Circ VRK1 siRNA significantly increased the tube formation capacity of the cells (Fig. 3B). Detection of VEGF expression using qPCR and western blot showed that the mRNA and protein expression of VEGF in cells was decreased significantly with OGD/R treatment, while Circ VRK1 downregulation significantly increased the VEGF expression in cells (Fig. 3C, D).

### Circ VRK1 knockdown attenuates brain injury in mice

**Fig. 4:**
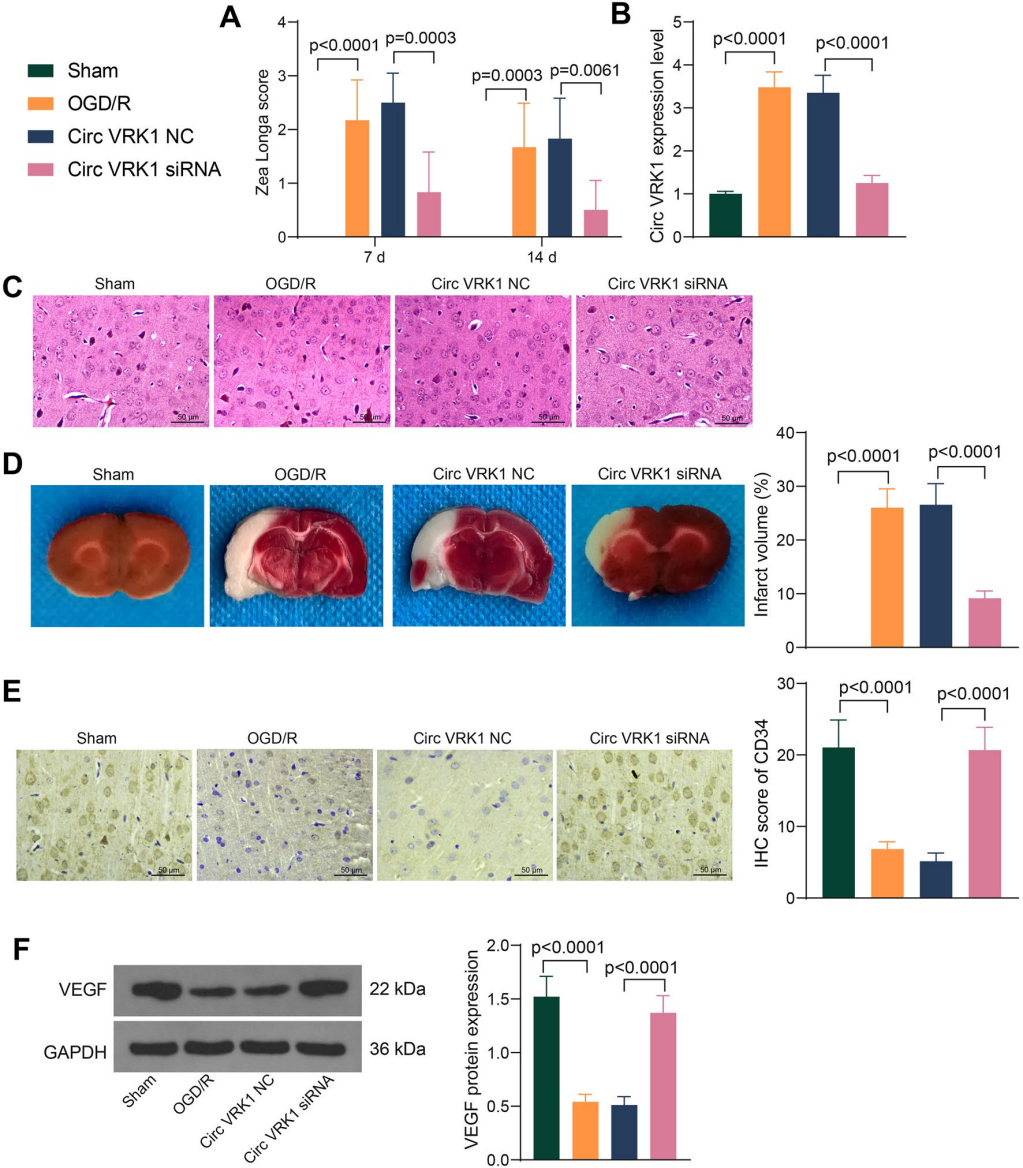
Silencing of Circ VRK1 alleviates brain injury in mice. C57BL/6 mice were modeled with OGD/R and injected with lentiviral vectors harboring Circ VRK1 NC or Circ VRK1 siRNA. **A** scoring of nerve damage in mice by Zea Longa scores. **B** the expression of Circ VRK1 in brain tissues of mice examined using PCR. **C** brain tissue damage in mice by HE staining. **D** The area of cerebral infarction in mice examined by TTC staining. **E** Score of CD34 IHC scores in tissues. **F** Representative pictures of western blotting for VEGF protein expression in brain tissues of mice, and all replicates are presented in the Additional file 1 (images of adequate length are not provided). Error bars represented the mean ± SD (n = 6). One-way (**B**, **D**) or two-way ANOVA (**A**) were used

For our evaluation of the role of Circ VRK1 in the mouse model, animals were divided into a Circ VRK1 siRNA-injected group and a control group injected with Circ VRK1 NC. All animals were scored on days 7 and 14 after anesthesia and ischemia/reperfusion. The sham-operated mice had no neurological deficits, while the mice in the OGD/R group had significant neurobehavioral deficits. Compared with the Circ VRK1 NC treatment, Circ VRK1 siRNA significantly reduced the neurobehavioral scores (Fig. 4A). We then examined the expression of Circ VRK1 in the brain tissues of mice using RT-qPCR. Higher expression of Circ VRK1 was observed in the OGD/R mice compared to the sham group, while Circ VRK1 siRNA significantly inhibited the expression of Circ VRK1 (Fig. 4B). The results of HE staining showed no obvious pathological changes in brain tissues in the sham-operated mice, with normal cell structure and uniform intercellular mass, and no edema appeared. By contrast, different degrees of cell damage, cytoplasmic edema, tissue interstitial and cell swelling were observed in the model mice. Downregulation of Circ VRK1 reduced the pathological changes and alleviated the edema in brain tissues (Fig. 4C). There was no white infarct in the brain tissues of the sham-operated mice in TTC staining result, and in the model group, the area of white infarct was mainly distributed in the blood supply area of the middle cerebral artery. However, the infarct volume was reduced after Circ VRK1 siRNA intervention (Fig. 4D). Therefore, Circ VRK1 siRNA intervention might reduce the incidence of cerebral infarction.

Subsequently, we assessed the expression of CD34 using IHC in mice. There were few CD34-positive cells in the sham-operated mice, and the IHC score of CD34 was appreciably decreased in the ischemic area of the model mice relative to the sham-operated mice. Downregulation of Circ VRK1 enhanced the IHC score of CD34 (Fig. 4E). Western blot detection revealed that VEGF was significantly downregulated in brain-injured mice and significantly upregulated after Circ VRK1 siRNA treatment (Fig. 4F). We demonstrated that Circ VRK1 downregulation alleviated the brain injury induced by ischemia/reperfusion in mice.

### Circ VRK1 mediates PTEN expression via sponging miR-17

**Fig. 5:**
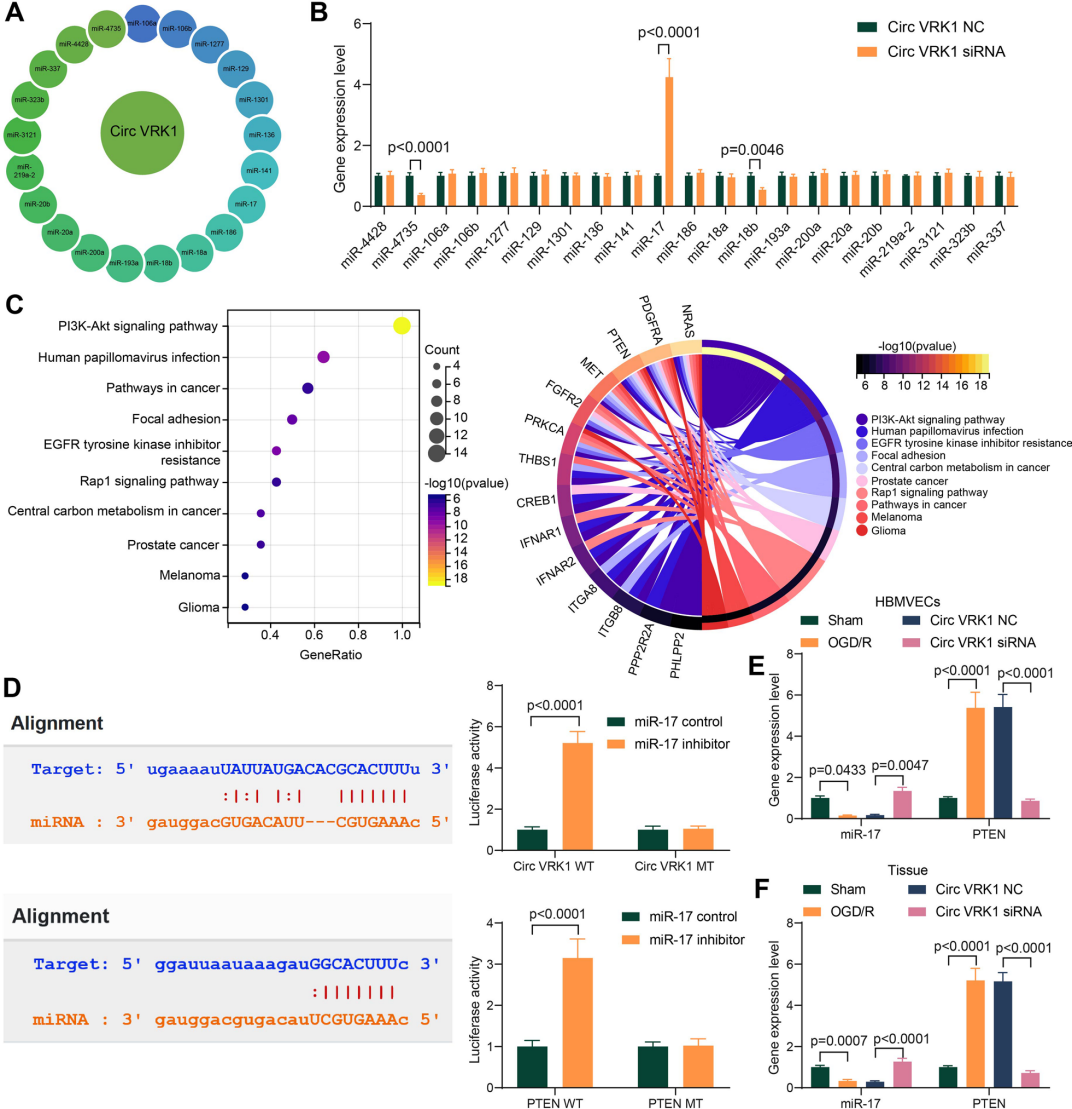
MiR-17 is the target of Circ VRK1, and PTEN is the target of miR-17 in HBMVECs. **A** Circ VRK1 downstream miRNAs predicted by StarBase. **B** miRNA expression changes in Circ VRK1 downregulated cells by qPCR. **C** KEGG pathway analysis of miRNA target genes in theStarBase. **D** The targeting relationship between Circ VRK1 and miR-17, miR-17 and PTEN verified by dual-luciferase assay. **E** miR-17 and PTEN mRNA expression in HBMVECs by qPCR. **F** miR-17 and PTEN mRNA expression in brain tissues of mice by RT-qPCR. Error bars represented the mean ± SD from 3 independent experiments or n = 6. Two-way ANOVA was used

The downstream miRNAs of Circ VRK1 were explored in the StarBase website (https://starbase.sysu.edu.cn/index.php) (Fig. 5A). PCR assays for miRNA expression in Circ VRK1 downregulated cells revealed that miR-17 was significantly upregulated in the cells (Fig. 5B). We then downloaded the downstream target genes of miR-17 in the StarBase website. Target genes were found to be significantly enriched to the PI3K/Akt pathway by KEGG pathway enrichment analysis, and PTEN is a key gene in the PI3K/AKT pathway (Fig. 5C). We speculated that miR-17 may mediate the PI3K/AKT pathway activity by targeting PTEN. The results of the bioinformatics prediction were verified by dual-luciferase assays, and the luciferase activity of both Circ VRK1 WT and PTEN WT were found to be significantly increased in cells transfected with miR-17 inhibitor (Fig. 5D). The expression of miR-17 and PTEN was examined in OGD/R-treated and Circ VRK1-downregulated cells. It was found that miR-17 was significantly downregulated, while PTEN was significantly upregulated in OGD/R-induced cells. However, Circ VRK1 siRNA significantly reversed the change in miR-17 and PTEN induced by OGD/R (Fig. 5E). Consistently, qPCR assay on brain tissues confirmed the expression changes in response to OGD/R and Circ VRK1 siRNA (Fig. 5F).

### **Silencing of miR-17 counteracts protective effect of Circ VRK1 siRNA on OGD/R-induced cell injury in HBMVECs**

**Fig. 6:**
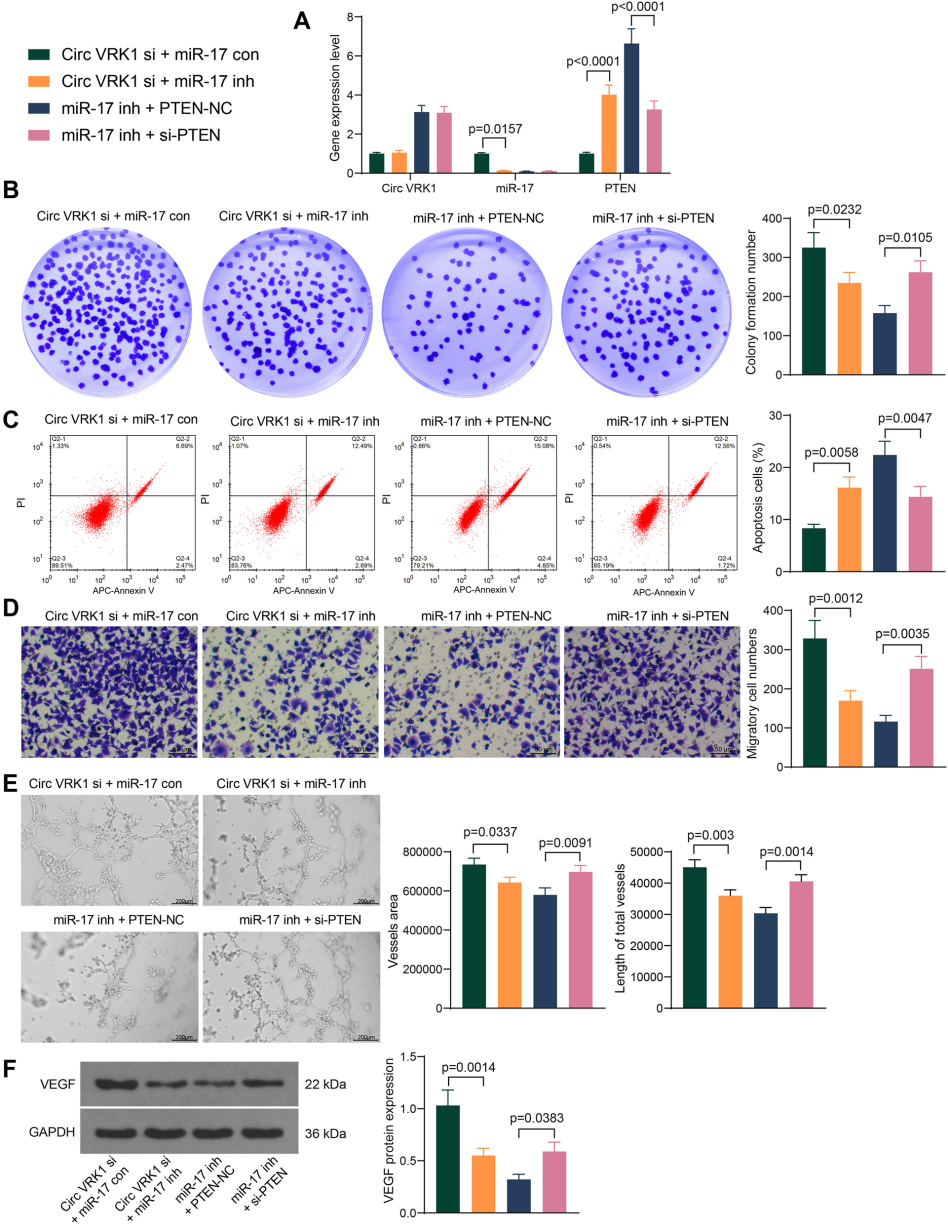
miR-17 inhibitor flattens the protection of Circ VRK1 siRNA on HBMVECs against OGD/R. HBMVECs were infected with lentiviral vectors harboring Circ VRK1 si + miR-17 con, Circ VRK1 si + miR-17 inh, miR-17 inh + PTEN-NC or miR-17 inh + si-PTEN and exposed to OGD/R. **A** Validation of lentiviral vector infection by qPCR. **B** proliferation of HBMVECs examined by colony formation assay. **C** apoptosis of HBMVECs examined by flow cytometry. **D** Migration of HBMVECs assessed by Transwell assay. **E** Tube formation of HBMVECs assessed by tube formation assay. **F** Representative pictures of western blotting for VEGF protein expression in HBMVECs, and all replicates are presented in the Additional file 1 (images of adequate length are not provided). Error bars represented the mean ± SD from 3 independent experiments. One-way (**B**–**F**) or two-way ANOVA (**A**) were used

The expression of Circ VRK1, miR-17 and PTEN was detected in the cells after simultaneous downregulation of Circ VRK1 and miR-17 as well as miR-17 and PTEN. It was found that miR-17 inhibitor increased PTEN expression in the cells in the presence of Circ VRK1 siRNA (Fig. 6A). Detection of the cellular activity of HBMVECs using colony formation assay revealed that miR-17 inhibitor diminished the proliferation, while miR-17 inh + si-PTEN enhanced the proliferation (Fig. 6B). Moreover, flow cytometry showed that miR-17 inhibitor aggravated apoptosis and si-PTEN alleviated apoptosis induced by miR-17 inhibitor (Fig. 6C). In addition, miR-17 inhibitor mitigated the pro-migratory and pro-angiogenic effects of Circ VRK1-si, while si-PTEN abrogated the anti-migratory and anti-angiogenic effects of miR-17 inhibitor (Fig. 6D, E). Finally, it was found that miR-17 inhibitor decreased VEGF expression and PTEN siRNA increased VEGF expression in the cells (Fig. 6F).

### Silencing of miR-17 counteracts protective effect of Circ VRK1 siRNA on mice

**Fig. 7:**
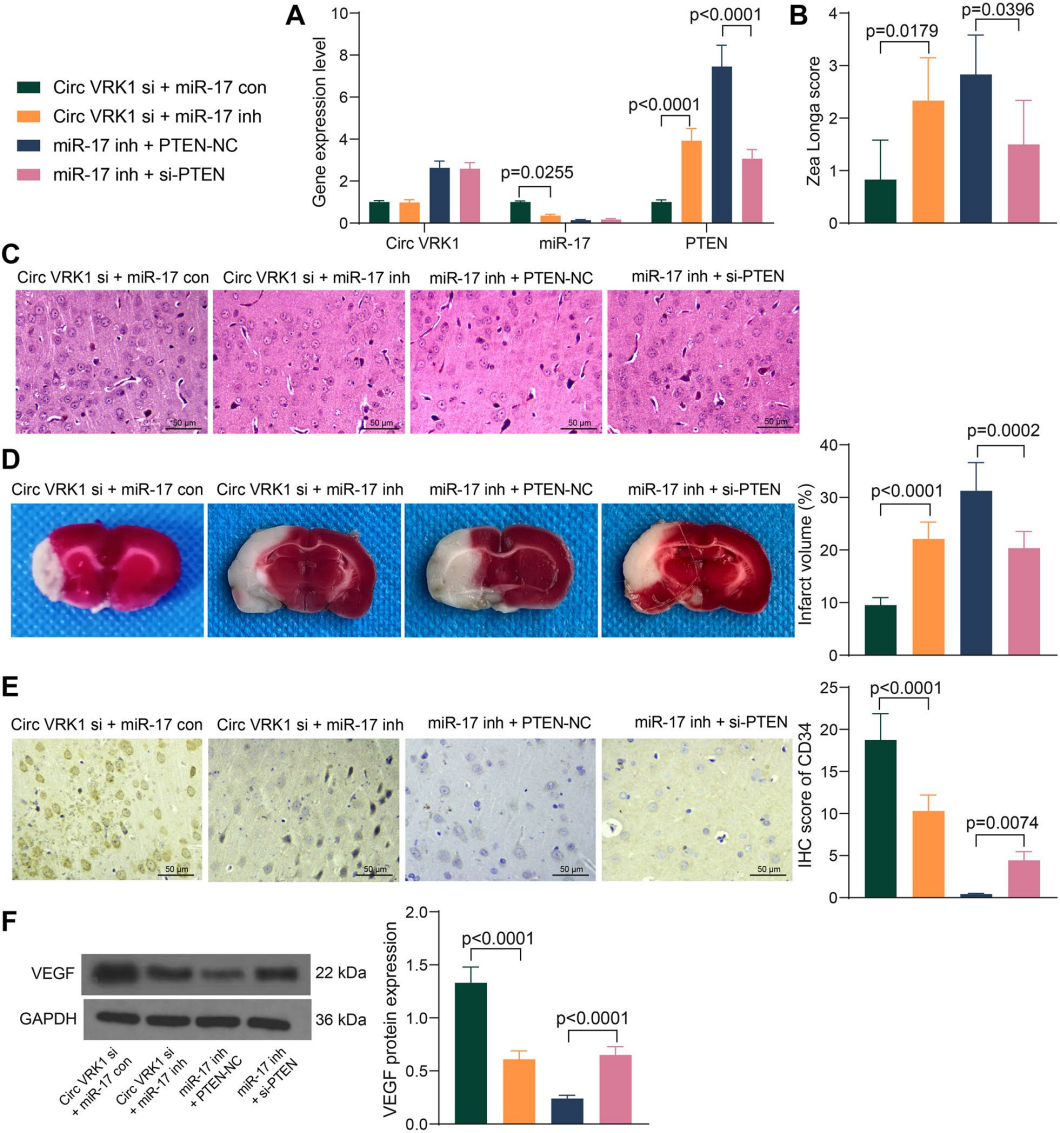
miR-17 inhibitor overturns the alleviating effects of Circ VRK1 siRNA on brain injury in mice. C57BL/6 mice with cerebral ischemia/reperfusion were injected with lentiviral vectors harboring Circ VRK1 si + miR-17 con, Circ VRK1 si + miR-17 inh, miR-17 inh + PTEN-NC or miR-17 inh + si-PTEN. **A** Circ VRK1, miR-17 and PTEN expression by PCR. **B** scoring of nerve damage in mice by Zea Longa scores. **C** brain tissue damage in mice by HE staining. **D** the area of cerebral infarction in mice examined by TTC staining. **E** IHC scores of CD34 in brain tissues. **F** Representative pictures of western blotting for VEGF protein expression in brain tissues, and all replicates are presented in the Additional file 1 (images of adequate length are not provided). Error bars represented the mean ± SD from 3 independent experiments. One-way (**B**, **D**, **F**) or two-way ANOVA (**A**) were used

Likewise, lentiviral vectors containing both Circ VRK1-si + miR-17 inhibitor and miR-17 inhibitor + si-PTEN were injected into mice. Successful delivery was confirmed by PCR (Fig. 7A). Inhibition of miR-17 aggravated neurological damage in mice, whereas PTEN downregulation partially restored neurological deficits (Fig. 7B). HE staining showed that miR-17 downregulation exacerbated brain tissue edema and neuronal morphological abnormalities induced by cerebral ischemia/reperfusion in mice, while PTEN silencing reduced brain tissue damage (Fig. 7C). Staining of brain tissues using TTC also revealed that miR-17 inhibitor induced an increase in brain infarct area in mice, while PTEN siRNA inhibited its effect and reduced the infarct area (Fig. 7D). IHC and western blot were conducted to assess the expression of CD34 and VEGF, respectively. It was exhibited that depletion of miR-17 lowered the expression of CD34 and VEGF, while PTEN siRNA enhanced the expression of both (Fig. 7E, F).

### PI3K/AKT pathway participates in the regulation of Circ VRK1/miR-17/PTEN axis on HBMVECs

**Fig. 8:**
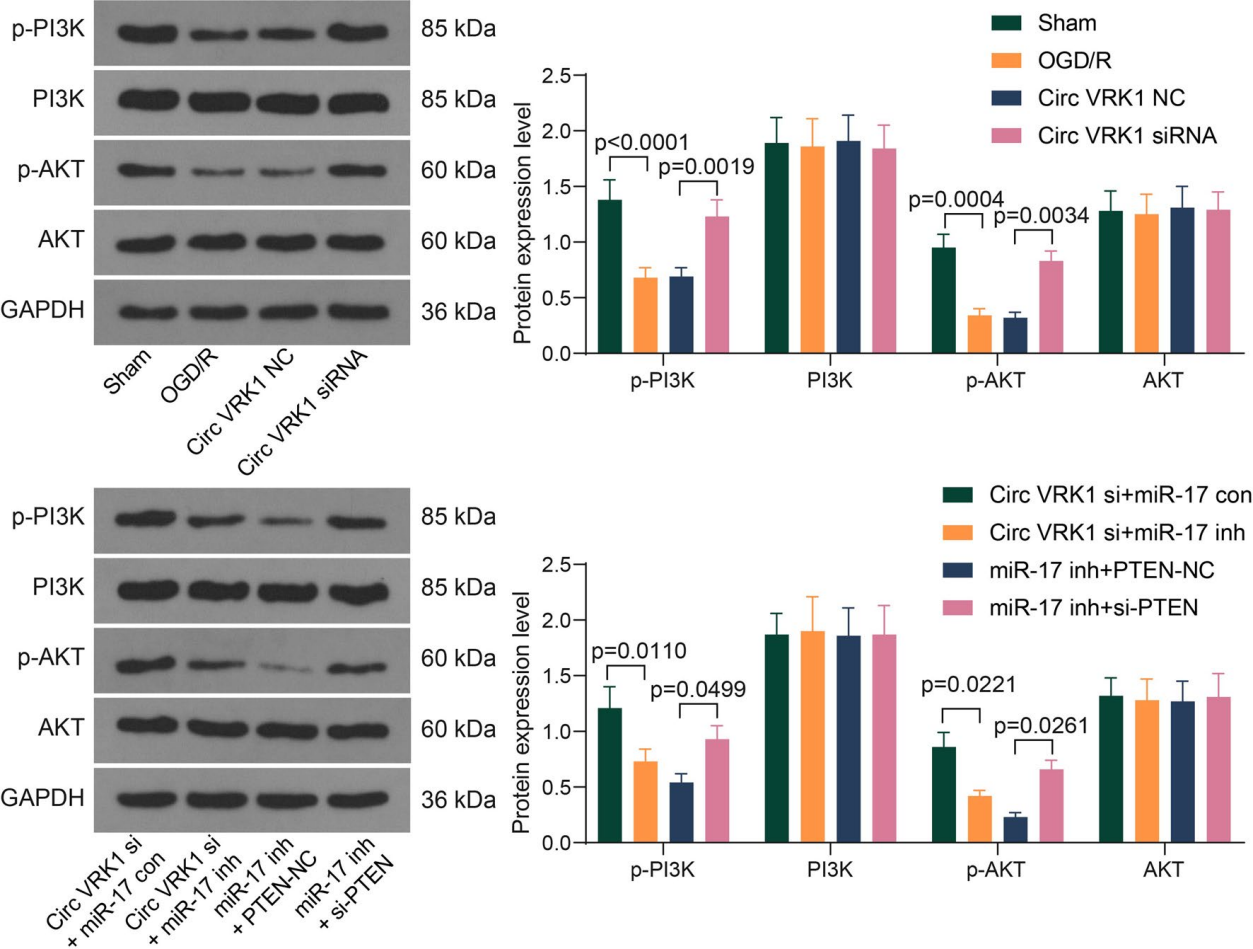
Silencing of Circ VRK1 activates the PI3K/AKT pathway by regulating miR-17/PTEN. Representative pictures of western blotting for p-PI3K, PI3K, p-Akt, and Akt in HBMVECs after different infection, and all replicates are presented in the Additional file 1 (images of adequate length are not provided). Error bars represented the mean ± SD from 3 independent experiments. Two-way ANOVA was used

To attest the reliability of bioinformatics analysis, we detected the changes of PI3K/AKT pathway activity, mainly the phosphorylation of PI3K and AKT in HBMVECs using western blot (Fig. 8). We found that the extent of PI3K and AKT phosphorylation was significantly decreased after OGD/R treatment, which was increased with Circ VRK1 downregulation treatment. miR-17 inhibitor repressed the effect of Circ VRK1 downregulation to block PI3K/AKT activity, while PTEN siRNA inhibited miR-17 downregulation to activate the PI3K/AKT signaling. This suggests that Circ VRK1/miR-17/PTEN axis regulates OGD/R in HBMVECs through mediating the PI3K/AKT pathway.

## Discussion

Cerebral ischemic diseases, including cerebral ischemia-reperfusion injury, can lead to serious dysfunction of the brain, which is associated with extremely high mortality and disability, and circRNAs are enriched in numerous organs, especially in the brain, which may be involved in cerebral physiological and pathological processes [16]. For instance, knockdown of circ_ANRIL elicited anti-apoptotic and anti-inflammatory effects by promoting the expression of miR-622 in HBMVECs exposed to OGD/R [17]. In the current study, we examined the role of Circ VRK1 in cerebral ischemia-reperfusion injury. Our data established that the expression of Circ VRK1 and PTEN was significantly upregulated in OGD/R-treated HBMVECs, while that of miR-17 was downregulated. Depletion of Circ VRK1 attenuated OGD/R-induced oxidative stress and inflammatory responses, while promoted angiogenesis and migration in HBMVECs. More importantly, the PI3K/AKT pathway was involved in the regulatory effect of Circ VRK1/miR-17/PTEN axis on OGD/R-induced cell injury.

A total of 9 significantly increased circRNAs has been found out during our microarray analysis, and Circ VRK1 was picked owing to the greatest expression foldchange among these 9 circRNAs. After cerebral ischemia/reperfusion, a large repertoire of circulating inflammatory cells infiltrate the injured area, which contributes to the release of inflammatory mediators and an inflammatory cascade that ultimately triggers secondary brain damage, including blood-brain barrier destruction, cerebral edema, and oxidative stress [18]. In the present study, Circ VRK1 knockdown was revealed to elicit the anti-inflammatory effects through blocking the activation of TNF-α, IL-1β, IL-6 in HBMVECs. Also, we tested the activity of SOD, the content of MDA, and intracellular ROS in the cells exposed to OGD/R. The results revealed that Circ VRK1 siRNA had an antioxidant effect. The suppressing effects of HBMVEC angiogenesis is positively linked to the survival of patients with stroke [19], which makes promoting HBMVEC angiogenesis a possible target for improving the prognosis of cardiovascular diseases. In the present study, we found that knockdown of Circ VRK1 promoted HBMVEC viability, migration, and angiogenesis. Moreover, we revealed that Circ VRK1 relieved brain tissue edema and enhanced CD34-positive cells in mice. Furthermore, through bioinformatics and KEGG pathway analyses, we found that miR-17/PTEN/PI3K/AKT axis might be the downstream effector of Circ VRK1. Previously, induction of miR-17-5p has been identified to protect the kidney against ischemia-reperfusion injury [20]. Moreover, the direct binding relation between miR-17 and PTEN has been substantiated in vascular smooth muscle cells and ovarian granulosa cells [21, 22], which was largely in agreement with our prediction and experimental results.

In addition, our in vitro evidence presented that miR-17 inhibitor mitigated the stimulating effects of Circ VRK1 siRNA on HBMVEC viability, migration and tube formation. By contrast, overexpression of miR-17-5p fostered angiogenesis in nasopharyngeal carcinoma [23]. Most importantly, miR-17-5p suppressed cerebral hypoxia/reoxygenation injury by binding to PTEN via the PI3K/AKT/mTOR signaling pathway [24]. In the current study, PTEN knockdown was revealed to rescue the repressive effects of miR-17 inhibitor both in vitro and in vivo. Likewise, PTEN overexpression reversed the pro-angiogenic effect of miR-139-5p overexpression in high glucose-treated mouse retinal microvascular endothelial cells, contributing to reduced cell migration and tube formation [25]. Furthermore, a PTEN inhibitor BPV could appreciably prevent myocardial infarction in mice via promoting cardiomyocyte angiogenesis and activating the PI3K/AKT/VEGF signaling pathway [26]. Also, long non-coding RNA FAL1 is able to protect HBMVECs against OGD/R-induced cell damage by regulating the PAK1/AKT signaling pathway [27]. Administration of PI3K inhibitors LY294002 and BKM120 impaired the effects of Vinpocetine, a semi-synthetic alkaloid derivative, on oxidative stress, inflammation, and apoptosis in brain tissues from rats with cerebral I/R injury [28]. Here, we observed that silencing of Circ VRK1 or PTEN activated the PI3K/AKT signaling, while miR-17 inhibitor impaired the PI3K/AKT signaling. This suggests that the PI3K/AKT signaling is a downstream signaling pathway regulated by Circ VRK1/miR-17/PTEN. Circ VRK1/miR-17/PTEN could mediate the PI3K/AKT signaling to engage in HBMVEC damage induced by OGD.

In conclusion, Circ VRK1 knockdown ameliorated oxidative stress and inflammatory response, while expedited angiogenesis of OGD/R-induced HBMVECs by sponging miR-17, a process involving PTEN/PI3K/AKT axis. These data displayed the crucial role of Circ VRK1 in OGD/R-induced cell damage in vitro and cerebral ischemia-reperfusion injury in vivo. The findings pave the way for future study and offer a novel strategy for further research on cerebral ischemia-reperfusion injury therapy. This study has some limitations that must be acknowledged. First, we analyzed gene expression assays using the 8th h samples based on the gradual upregulation of Circ VRK1 as the OGD exposure prolongs. However, the outlier cells might have survived the insult at the 8th h. Therefore, reconducting gene expression assays on the 2nd h are necessary to support our conclusion. Second, considering the complex microenvironments and the interaction of PI3K/AKT pathway with other miRNAs, we would further make investigations on the regulation of other miRNAs on the PI3K/AKT pathway during cerebral ischemia-reperfusion injury.

## Supplementary Information

**Additional file 1:** Three replicates of western blot images.

## Data Availability

All data generated or analyzed during this study are included in this published article.

## Abbreviations

APC: Allophycocyanin
CCK8: Cell counting kit-8
circRNAs: circular RNAs
DCFH-DA: Dichloro-dihydro-fluorescein diacetate
DMEM: Dulbecco's modified Eagle's medium
ECA: External carotid artery
ELISA: Enzyme-linked immunosorbent assay
FBS: Fetal bovine serum
GAPDH: Glyceraldehyde-3-phosphate dehydrogenase
HBMVECs: Human brain microvascular endothelial cells
HE: Hematoxylin-eosin
KEGG: Kyoto Encyclopedia of Genes and Genomes
MCA: Middle cerebral artery
miRNA: microRNA
OD: Optical density
OGD/R: Oxygen-glucose deprivation/reoxygenation
RIPA: Radio immunoprecipitation assay
TTC: Tripheny tetrazolium chloride
