# Supplementary figures and images for: Circ VRK1/microRNA-17/PTEN axis modulates the angiogenesis of human brain microvascular endothelial cells to affect injury induced by oxygen-glucose deprivation/reperfusion
Source: BMC Neurosci. 2023 Jan 27;24:8. doi: 10.1186/s12868-023-00774-8 (PMC9881374; doi:10.1186/s12868-023-00774-8)

## full-length gels and blots

Fig 3D

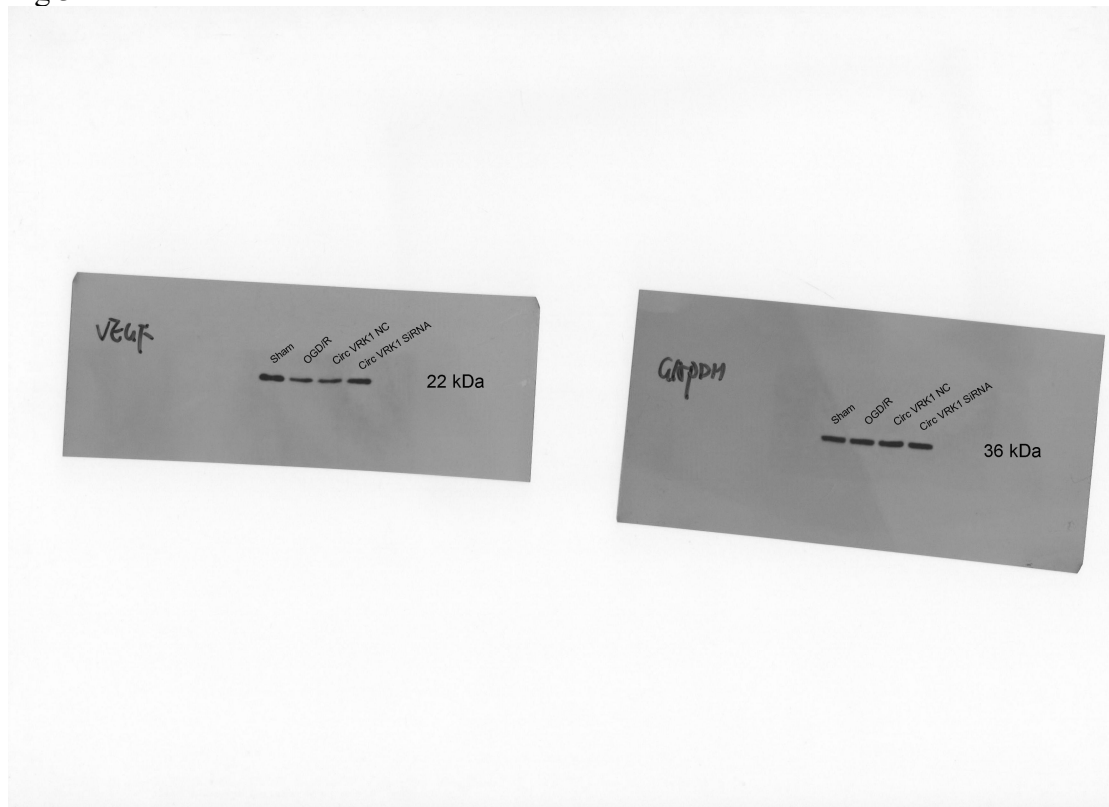

Fig 4F

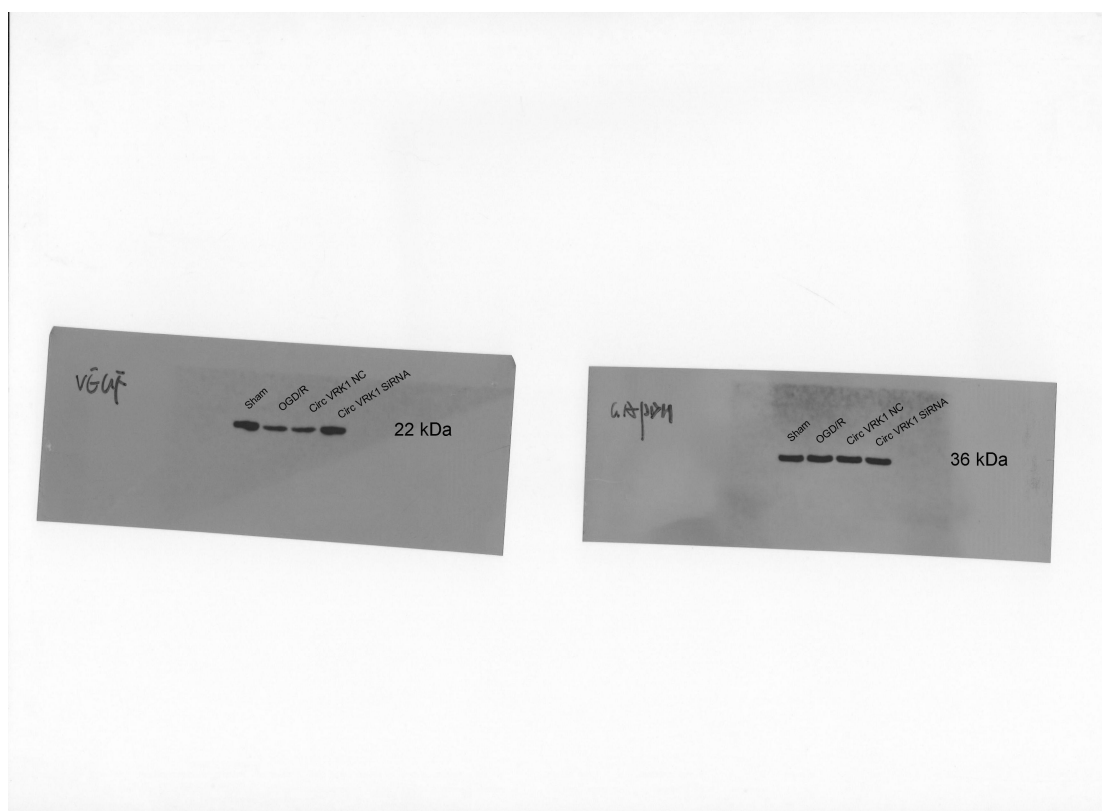

Fig 6F

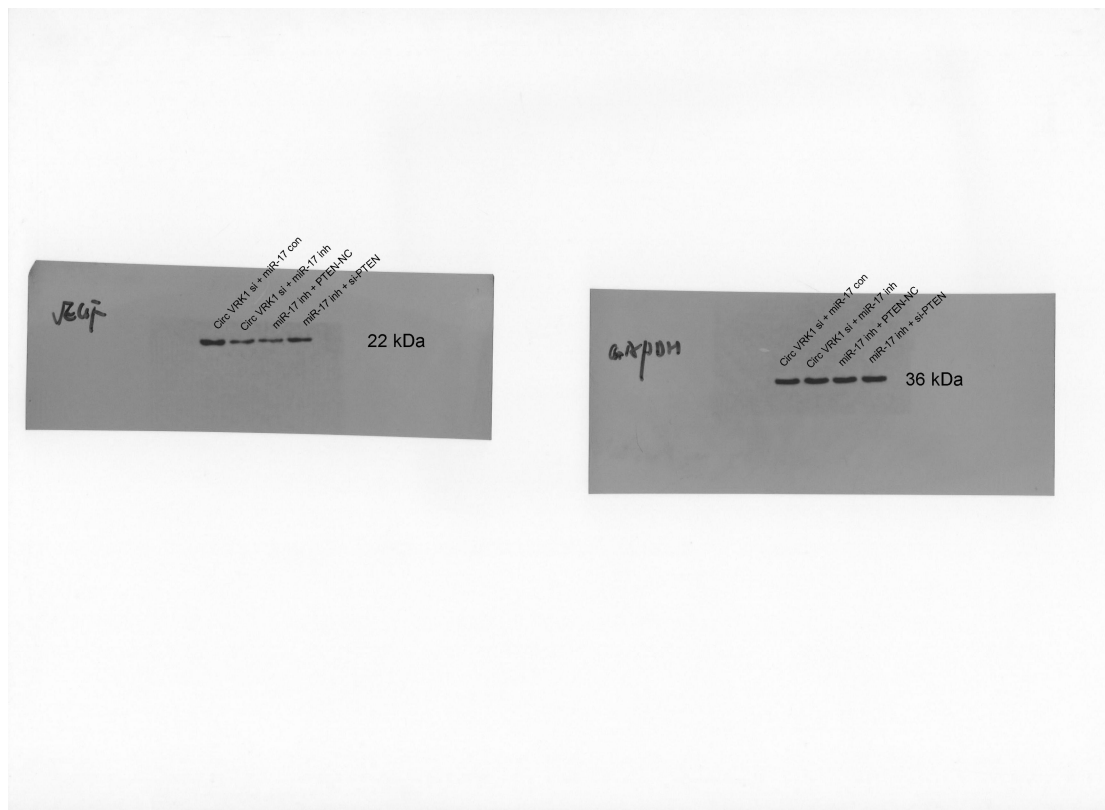

Fig 7F

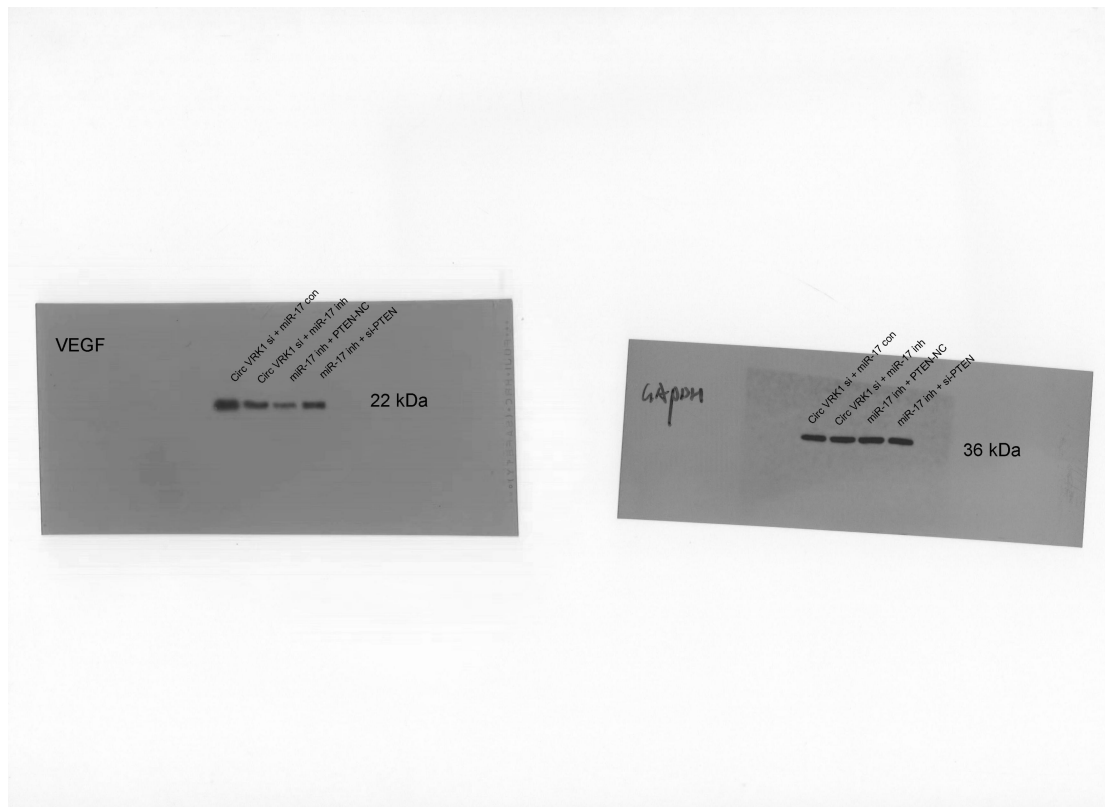

Fig 8-1

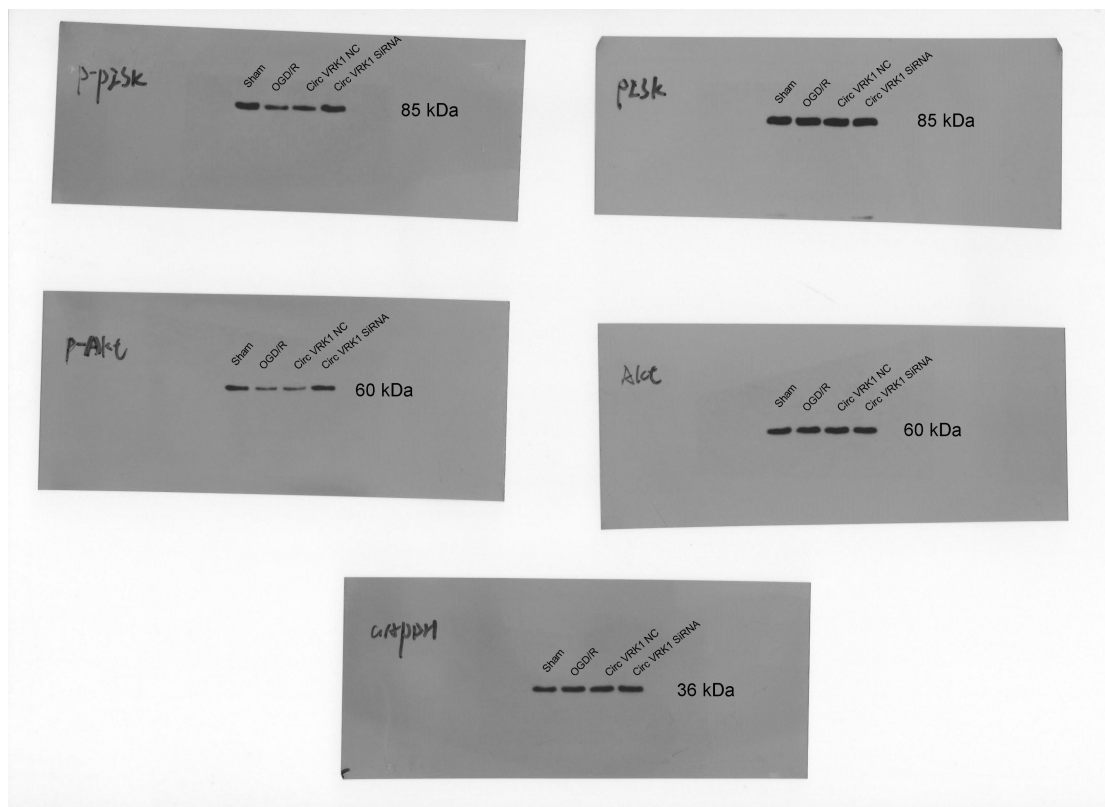

Fig 8-2

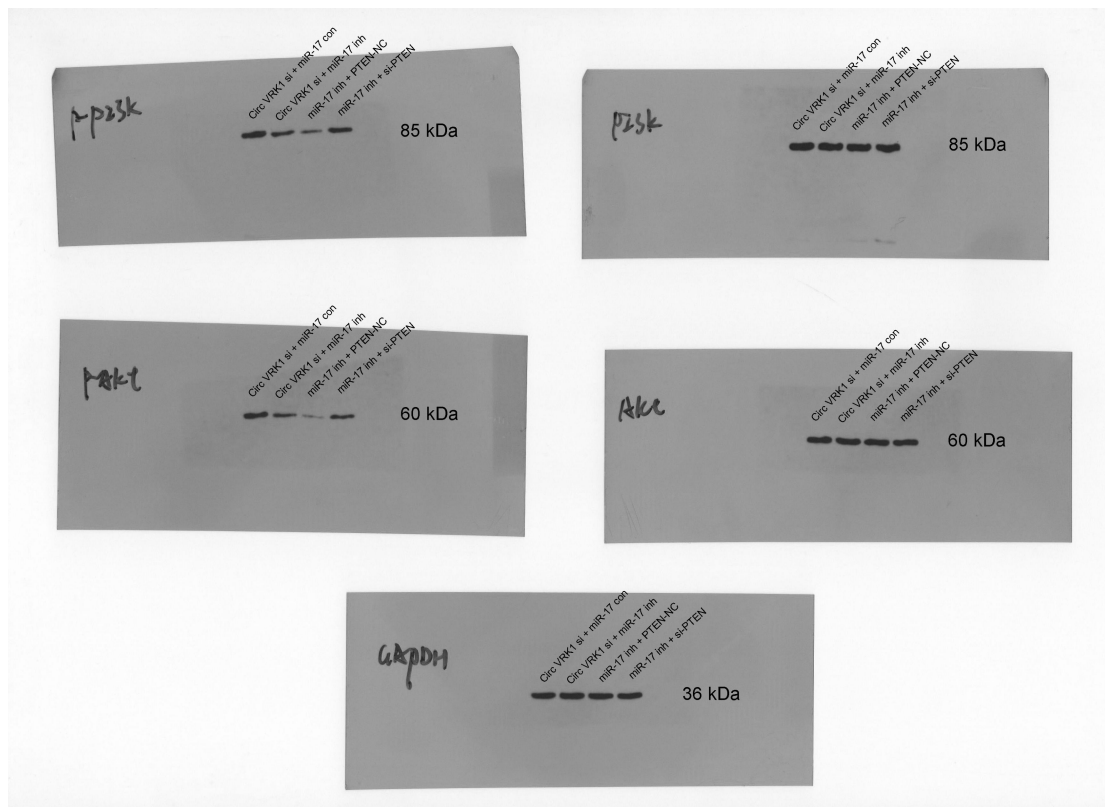

Supplement: Supplementary file 1 — Additional file 1: Three replicates of western blot images. [file 12868_2023_774_MOESM1_ESM.pdf]
